# Effective Management of McKenzie Derangement VI With Urge Urinary Incontinence Through Surgical Intervention and Customized Physiotherapy: A Case Report

**DOI:** 10.7759/cureus.70610

**Published:** 2024-10-01

**Authors:** Gurjeet Kaur, Nikita Gangwani, Subrat N Samal

**Affiliations:** 1 Physiotherapy, Datta Meghe Institute of Higher Education and Research, Wardha, IND; 2 Musculoskeletal Physiotherapy, Ravi Nair Physiotherapy College, Datta Meghe Institute of Higher Education and Research, Wardha, IND

**Keywords:** low-back pain (lbp), mckenzie derangement, prolapsed intervertebral disc (pivd), radiating pain, surgical spinal decompression, urge urinary incontinence

## Abstract

Urge incontinence, often linked to prolapsed intervertebral disc (PIVD) due to nerve compression, involves sudden, involuntary urine leakage. Management includes behavioral therapy, bladder training, and pelvic floor muscle training (PFMT) to strengthen pelvic muscles and regulate voiding, effectively reducing symptoms and improving quality of life. A 40-year-old female patient presented with chief complaints of lower back pain radiating down the right lower limb below the knee, accompanied by a right-sided listing. Based on the McKenzie classification, she was diagnosed with McKenzie derangement VI, characterized by unilateral/asymmetrical pain across the L4-L5 region, radiating pain below the knee, and the presence of deformity, specifically trunk deviation away from the painful side that is the right side. Additionally, she reported experiencing urge urinary incontinence. Following surgery, she experienced significant reductions in pain and urinary incontinence, improved posture, and an enhanced quality of life. Surgical decompression and spinal fixation effectively alleviated neural compression and instability at the L4-L5 and L5-S1 levels. At the same time, the phased physiotherapy approach facilitated recovery through pain management, neuromuscular re-education, and functional restoration. Preoperative assessments such as the modified Schober’s test, visual analogue scale (VAS), Revised Oswestry Disability Index (RODI), sciatica bothersome index, revised urinary incontinence scale (RUIS), and Michigan incontinence symptom index (MISI) were essential in guiding the rehabilitation process and measuring progress. The integration of these multidisciplinary interventions underscores the importance of a comprehensive treatment plan in achieving positive outcomes for PIVD, despite the potential for complications such as chronic pain and disability.

## Introduction

Prolapsed, herniated, or displaced intervertebral disc is a frequently observed medical condition [1]. The prolapsed intervertebral disc (PIVD) is a specific medical disorder in which the posterior longitudinal ligament ruptures and disc material protrudes into the spinal canal. Symptoms are more likely caused by subtle microtrauma to neural tissue due to spinal instability rather than direct neural compression or deformation [2]. The intervertebral disc consists of the annulus fibrosus, a dense ring of collagen surrounding the nucleus pulposus [3]. Disc herniation occurs when part or all of the nucleus pulposus protrudes through the annulus fibrosus. The primary cause is degeneration, where the nucleus pulposus loses hydration and strength as it ages [4]. The annual incidence of herniated discs is approximately five to 20 cases per 1,000 adults, occurring most frequently in individuals between their 30s and 50s, with a male-to-female ratio of 2:1 [5]. Herniated discs often follow an injury, causing sharp, radiating pain along the affected nerve root. Patients may also experience numbness, tingling, reduced sensation, and, in severe cases, weakness or instability while walking [6].

A herniated lumbar disc can cause sensory and motor deficits specific to the affected nerve root, with pain often radiating to the thigh, leg, or foot. Key history includes symptom onset, pain location, and previous treatments [7]. Also, incontinence is an obvious symptom, making it crucial to inquire about it, especially when urge incontinence is associated with radiculopathy [8]. Over 85% of acute herniated disc cases resolve within eight to 12 weeks without specific treatment, but abnormal neurological exams or refractory cases require further evaluation. X-rays assess structural instability, and computed tomography (CT) scans visualize bones and calcified discs. At the same time, magnetic resonance imaging (MRI) is the most sensitive method for detecting herniated discs and guiding treatment [9]. Also listing can be seen in the case of PIVD. Listing in PIVD refers to a lateral to relieve nerve compression that moves the trunk away from the side of the prolapsed disc. This shift causes a visible deviation of the spine from the midline, resulting in an altered posture. The patient may appear asymmetrical, with one shoulder higher than the other. Listing is a compensatory mechanism to reduce pain and nerve irritation [10]. She was diagnosed with Mckenzie derangement VI, marked by unilateral or asymmetrical pain in the L4-L5 region, radiating pain below the knee, and a deformity characterized by trunk deviation away from the painful side [11]. Acute lumbar radiculopathies are primarily managed with nonsteroidal anti-inflammatory drugs (NSAID) and delayed physical therapy, with opioids and epidural injections as second-line options for severe or persistent cases. Surgery, such as laminectomy with discectomy and posterior decompression, is reserved as a last resort, offering moderate but diminishing long-term benefits [12]. A herniated disc might cause complications, including chronic back pain, permanent nerve damage, and economic impacts like work loss and disability. Most discectomies are successful, though some require repeat surgery [13]. In this case report, the patient underwent posterior decompression surgery at the L4-L5 and L5-S1 levels, along with spinal fixation at L4-L5, to alleviate nerve compression and stabilize the spine. Following surgery, the patient participated in a tailored physiotherapy program designed with a structured protocol. This physiotherapy focused on pain reduction, enhancing spinal mobility, and progressively restoring functional capacity, posture and improving urge urinary incontinence (UUI). The tailored approach focused on exercises and therapies suited to the patient's individual needs, leading to notable enhancements in her daily activities, mobility, and overall quality of life.

## Case presentation

Patient’s information

A 40-year-old female reported significant lower back pain and tingling in her left lower leg. She was in good health until one year ago when she sustained a fall on a slippery floor, landing on her back. Following the fall, she sought medical attention from a local doctor for her back pain. An X-ray was performed, but no abnormalities were detected. She was prescribed analgesics and advised to take bed rest, which provided relief after one month of treatment.

However, six months later, while bending down to perform household chores, she experienced a sudden sharp, shooting pain upon standing. From that point on, she began experiencing persistent pain during forward and side bending. Over time, she also developed intermittent tingling in the posterior aspect of her right lower limb, which progressively worsened, especially with prolonged sitting, standing, or walking. The patient also noticed a change in her posture due to compensation, as weight-bearing on the left side was painful. She observed that her right pelvis is elevated compared to the left side, and her trunk is leaning toward the right side to reduce pain. Additionally, she reported episodes of urge urinary incontinence, which emerged alongside these symptoms. The timeline of events related to the patient’s condition is presented in Table 1.

**Table 1:** Timeline of events

S no.	Timeline	Symptoms	Investigations	Management
1.	1 year ago	Severe lower back pain that began following a fall on a slippery surface	X-ray: no abnormalities detected	Prescribed analgesics and advised bed rest
2.	1 month later	Significant relief from back pain following bed rest and analgesics	None	Continued rest and analgesic treatment
3.	6 months later	Sudden sharp, shooting pain upon standing after bending. Persistent pain during forward and side bending	Clinical examination	Symptomatic treatment and advice on activity modification
4.	1 year later	Worsening tingling in the left lower limb, particularly with prolonged sitting, standing, or walking	MRI (suggested): evaluate nerve compression	Physiotherapy for posture correction and pain management
5.	3 months ago	Postural changes include elevation of the right pelvis and a right trunk lean resulting from pain	Postural assessment	Tailored physiotherapy program, ergonomic advice
6.	3 months ago	Episodes of urge urinary incontinence	Patients complain	Multidisciplinary approach including urology referral

Clinical presentation

The patient gave informed consent before her examination, with a clear understanding of the procedures involved. During the assessment, she was alert, oriented to time, place, and person, and exhibited clear cognitive function. Her cooperation and engagement throughout the process allowed for a thorough evaluation. Essential indicators such as heart rate, blood pressure, respiratory rate, and temperature, were stable, showing no signs of immediate physiological distress.

Physically, the patient presented with an ectomorphic body type, characterized by a slim, delicate build, with a body mass index (BMI) of 18.61 kg/m², categorizing her as underweight. This assessment occurred before her scheduled surgery. Observational findings included a Foley catheter, indicating ongoing management of urinary function.

During the clinical examination, significant muscle spasm was noted in the paraspinal muscles, likely due to pain or discomfort. Additionally, tightness was observed in the hip flexors, which may be contributing to restricted mobility. The patient exhibited a limited range of motion in the lumbar spine, with forward flexion notably aggravating her pain. On the visual analogue scale (VAS), she reported a pain level of 7.5, indicating moderate to severe discomfort. Lower back mobility was further evaluated using the modified Schober’s test, and the strength of her lumbar flexors and extensors was assessed via manual muscle testing, providing insight into her functional capacity.

During ambulation, the patient displayed marked difficulty rising from a chair and had a noticeable rightward trunk shift as she walked into the consulting room. This lateral shift deformity was verified upon inspection, and a reduction in lumbar lordosis was also observed. Although her pain severely limited movements in all directions, including right lateral flexion and right side gliding in the standing position was unaffected. Despite these limitations, the patient could walk on her toes and heels, her patellar and Achilles tendon reflexes were symmetrical and present, indicating complete neurological function.

**Figure 1:**
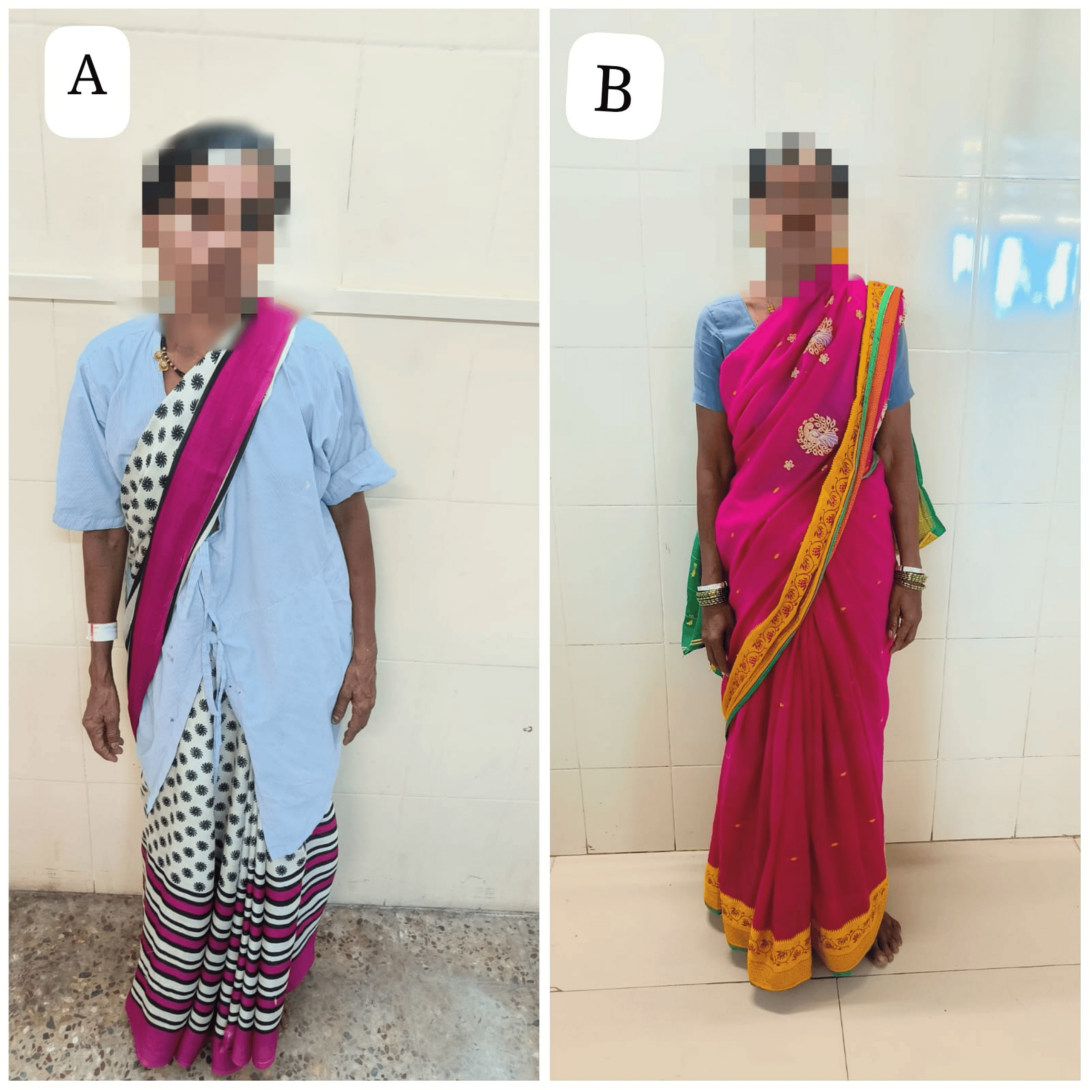
Posture assessment (A) The woman shows an uneven posture including elevation of the right pelvis and a right trunk lean resulting from pain. (B) Her posture is upright and balanced, reflecting significant improvement due to surgical management or rehabilitation

This comprehensive assessment highlights the interplay between her musculoskeletal symptoms, pain, and functional limitations, underscoring the need for a carefully tailored treatment plan. Figures 1A-1B show the posture of the patient before and after surgery.

On investigation

**Figure 2:**
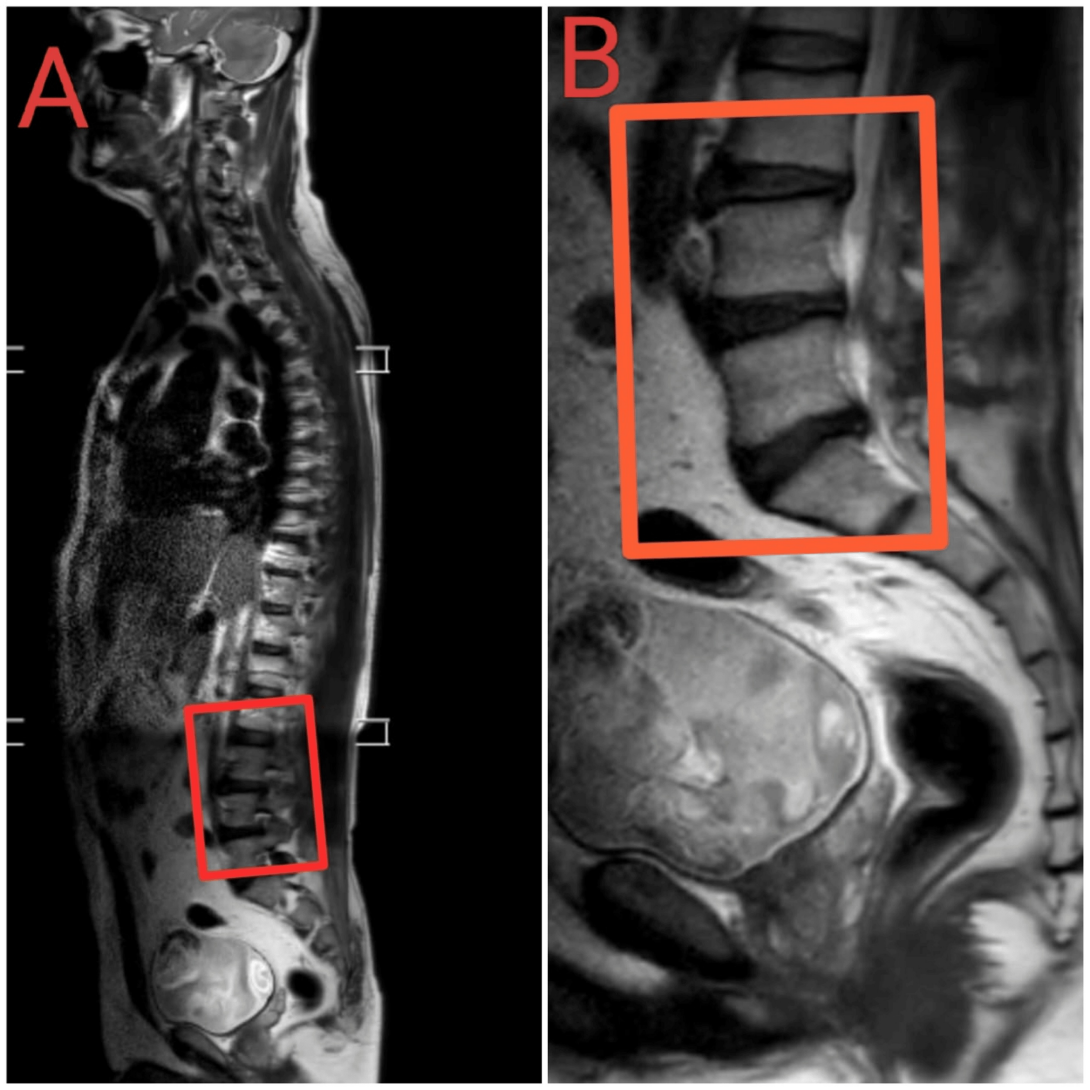
Presurgical magnetic resonance image This presurgery MRI image depicts sagittal views of the lumbar spine. The right image (A) shows the full length of the spine, while the left (B) image focuses on the lower lumbar region, specifically highlighting the L4-L5 and L5-S1 intervertebral discs

Prior to the surgery, the patient underwent comprehensive diagnostic evaluations, including a complete blood count (CBC), kidney function test (KFT), and liver function tests (LFT). All results were within normal limits, indicating no systemic health issues that could interfere with the surgical procedure or her knee condition. Additionally, MRI identified disc herniation at the L4-L5 and L5-S1 levels. These preoperative assessments were crucial for establishing an accurate diagnosis and ensuring the best possible surgical outcome. Figures 2A-2B denote the MRI in sagittal view. This was taken before the surgical procedure.

Surgery details

**Figure 3:**
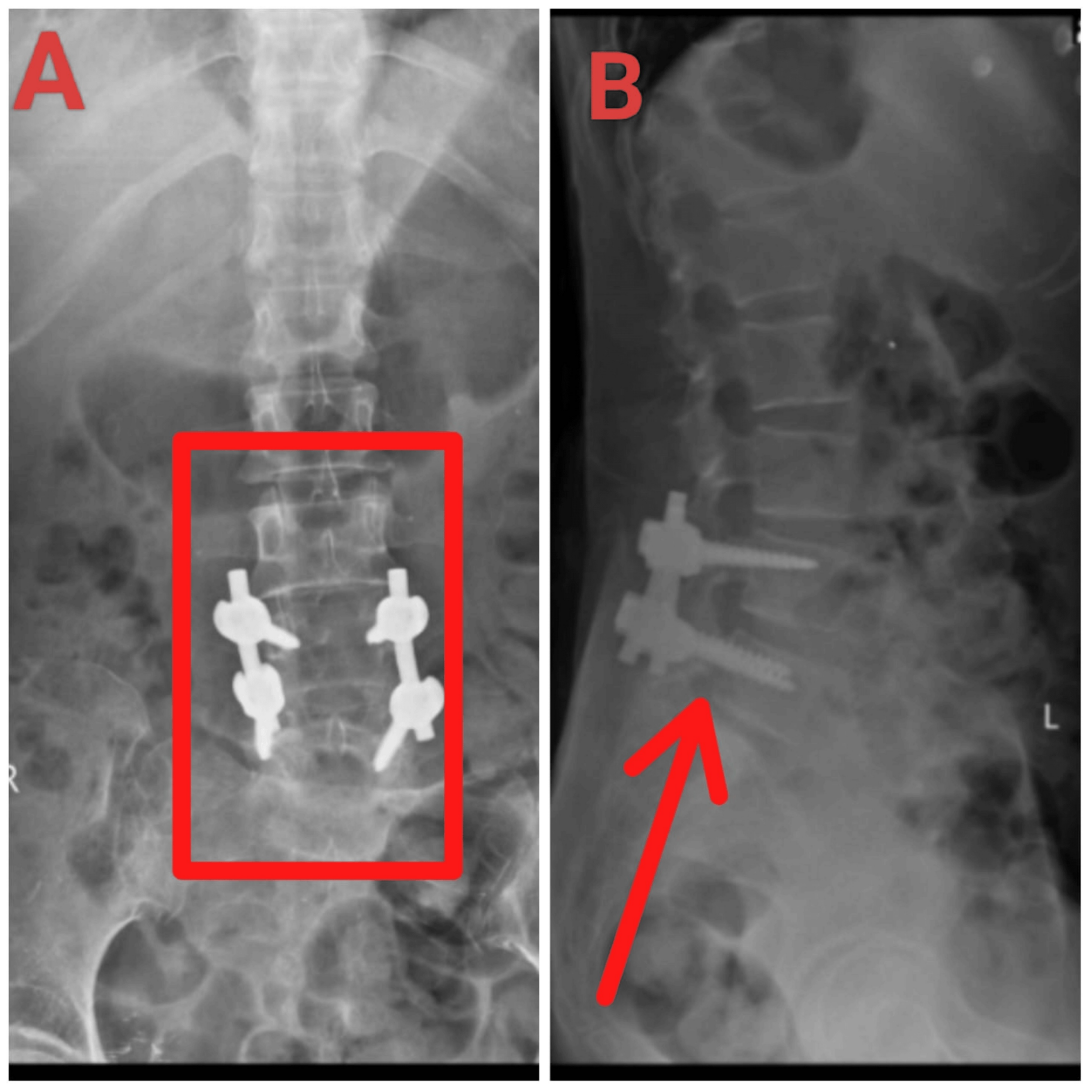
Postsurgery X-ray image (A) Anteroposterior (AP) view: The AP view complements this by offering a frontal perspective, showing the symmetric alignment of the screws and rods, indicating proper surgical placement. (B) Lateral view: The lateral view shows the pedicle screw and rod fixation hardware at the L4-L5 level, providing a side view that allows for the evaluation of spinal alignment and hardware placement in the sagittal plane

The patient underwent posterior decompression at L4-L5 and L5-S1, with spinal fixation at L4-L5 under general anesthesia. A 7 cm midline incision was made, and pedicle screws were inserted into L4 and L5, followed by connecting rods. Decompression and laminectomy addressed severe canal stenosis and ligamentum flavum hypertrophy at L4-L5 and L5-S1. The procedure concluded with thorough irrigation, layered closure with a drain, and an uneventful recovery. Implants included four polyaxial pedicle screws (5.5 x 35 mm) and two connecting rods (5.5 x 50mm, Jayon). Figures 3A-3B show the postoperative X-ray.

Diagnostic assessment

In the preoperative evaluation, the patient's lumbar range of motion was examined in a standing position using the modified Schober’s test, which measures the flexibility and mobility of the lumbar spine. For assessing back muscle strength, the patient was positioned prone, allowing for a focused evaluation of the paraspinal and other supporting muscles. Pain levels were determined at the time of assessment using the VAS, providing a subjective measure of pain intensity. To gauge functional outcomes, the Revised Oswestry Disability Index (RODI) and sciatica bothersome index were employed, offering insights into the patient’s daily functional limitations and the severity of sciatica symptoms. The revised urinary incontinence scale (RUIS) and Michigan incontinence symptom index (MISI) were used to check the type and severity of urinary incontinence. These comprehensive assessments informed the clinical decision-making process and provided baseline data for postoperative comparisons to evaluate recovery and treatment effectiveness. Table 2 depicts the range of motion of lumbar joint.

**Table 2:** Range of motion Modified Schober’s test was used. N/A: cannot assess due to pain

Joint and movement	Preoperative	Postoperative (day 1)	Post rehabilitation (week 14)
Forward flexion	3 cm	2 cm	8 cm
Extension	2 cm	N/A	6 cm

Table 3 provides a comprehensive analysis of the strength of lumbar joints, focusing on the muscle groups involved in spinal stability and mobility. The data illustrates the degree of muscle power in the lumbar region, emphasizing any deficits or asymmetries. These findings are instrumental in guiding the tailored physiotherapy approach.

**Table 3:** Manual muscle testing Grade 0: No detectable muscle contraction; grade 1: trace contraction present but no movement; grade 2: weak (partial range of motion achieved); grade 3: moderate (full range of motion completed); Grade 4: strong (full range reached with slight wavering or effort); grade 5: normal (back extensors reach and hold end position effortlessly)

Joint and movement	Preoperative	Postoperative (day 1)	Postrehabilitation (week 14)
Lumbar flexors	1/5	2/5	4/5
Lumbar extensors	1/5	2/5	4/5

Table 4 presents the strength of the lower limb joints.

**Table 4:** Manual muscle testing of bilateral lower limb

Joint and movement	Preoperative	Postoperative (day 1)	Postrehabilitation (week 14)
Hip			
Flexors	2/5	2-/5	4-/5
Extensor	2/5	2-/5	4/5
Abductors	3/5	2/5	4/5
Adductors	3/5	2/5	4/5
Knee			
Flexors	3+/5	3/5	4/5
Extensors	3+/5	3/5	4/5
Ankle			
Dorsiflexors	3+/5	3+/5	5/5
Plantar flexors	3+/5	3+/5	5/5

Physiotherapy protocol

Phase 1

Phase 1, the immediate postsurgical phase (week 0-4), focuses on reducing pain, promoting wound healing, increasing activity tolerance, and educating patients on body mechanics. Precautions include avoiding excessive tissue stress and lumbar spine movements. Treatment involves basic postop exercises and gradual aerobic activity increases. Table 5 details the initial postoperative physiotherapy protocol, emphasizing pain management and early mobility exercises.

**Table 5:** Phase 1 physiotherapy protocol HEP: Home exercise protocol; ADL: activity of daily living

Phase 1: Immediate postsurgical phase	Time frame	Goals	Precautions	Treatment summary	Dosage	Criteria for progression
	Week 0-week 4	1. Reduces pain and inflammation	1. Prevent excessive flexion and initial mobility or stress on tissues	Education on bed mobility and transfers	-	Pain and swelling within tolerance
		2. Increase activity tolerance		Basic postop exercises: ankle pumps, quadriceps exercises, diaphragmatic breathing, relaxation exercises, abdominal isometrics	10 x 3 sets	Independent HEP, back strengthening exercises in supine lying, core strengthening, etc.
		3. Encourage wound healing				Tolerate 15 min of exercise and 15-30 min of cardiovascular activity
		4. Increase aerobic tolerance	2. Avoid lifting, twisting, or bending the lumbar spine for 6 weeks			Functional ADL for self-care/hygiene
		5. Monitor for infection		Increase walking tolerance to ½ mile daily	15 minutes	
		6. Educate on body mechanics and posture for bed mobility		Reinforce sitting, standing, and ADL changes with good body mechanics	-	
		7. Improve bladder control		Kegels exercise	10 x 3 sets	

Phase 2

Phase 2 of rehabilitation focuses on re-establishing neuromuscular control, normalizing flexibility and gait, and improving functional tolerances. Precautions include avoiding lumbar loading, twisting, bending, and lumbar extension. Treatment includes back education, manual therapy, pelvic stabilization exercises, and cardiovascular training. Progress is measured by improved body mechanics, co-contraction ability, and increased exercise tolerance. Table 6 outlines the second phase of physiotherapy management, focusing on progressive strengthening and flexibility exercises.

**Table 6:** Phase 2 physiotherapy protocol ROM: Range of motion

Phase 2: Initiation of outpatient physiotherapy	Time frame	Goals	Precaution	Treatment protocol	Dosage	Criteria for progression
	4-8 weeks (2-3 times/week)	Re-establish neuromuscular recruitment of the multifidus	Avoid lumbar loading	Back education program	-	Knowledge of body/lifting mechanics
		Normalize flexibility deficits	Avoid twisting and bending	Manual therapy: joint mobilization (grade 1 or 2), scar tissue mobilization	-	Co-contraction of multifidus/transverse abdominals for 60 sec
		Normalize gait deviations	Limit lumbar extension	Exercises: train neutral lumbar position, diaphragmatic breathing, neural mobilization, pelvic stabilization, abdominal drawing-in maneuver, progressive stabilization and mobility exercises, unloaded pelvic/lumbar ROM, hip/knee flexibility exercises	-	Cardiovascular tolerance of 30 min/day
		Improve positional tolerances for work	No standing ROM testing for 12 weeks	Initiate cardiovascular training like walking and cycling		Dynamic sitting and standing tolerance of 15-60 min
		Improve posture	-	McKenzie exercise program for back and to correct listing	10 x 3 repetitions with holds	
		Improve balance	-	Balance exercises and modalities for symptom modulation as needed	-	

Phase 3

Phase 3 (8-14 weeks) focuses on advanced strengthening, flexibility, and stabilization exercises. Key components include manual therapy, proper lifting techniques, and activity-specific training. Progression criteria involve functional muscle strength, trunk range of motion, and exercise independence, while avoiding overstrain and maintaining proper body mechanics. Table 7 details the phase 3 physiotherapy protocol, emphasizing advanced stabilization and activity-specific training.

**Table 7:** Phase 3 rehabilitation protocol ROM: Range of motion; MMT: manual muscle testing

Phase 3:	Time frame	Goals	Precaution	Treatment summary	Criteria for progression
	8-14 weeks (2-3 times/week)	1. Continue with strengthening and flexibility exercises	Avoid excessive loading on the lumbar spine during lifting and posture training	1. Manual therapy: joint mobilization of thoracic spine, hip/pelvis restrictions, soft tissue mobilization	1. MMT within functional limits
		2. Initiate lifting and posture training	Maintain proper body mechanics during all exercises to prevent re-injury	2. Exercises: continue ROM exercises, advanced balance, neural mobilization, multiplane stabilization/mobility, advanced stabilization and proprioceptive training, advanced hip/core strengthening, lifting training with proper posture, and body mechanics drills	2. Independent with exercise program
		3. Progress stabilization and trunk control	Monitor for signs of overtraining or strain during advanced stabilization and cardiovascular activities	3. Activity-specific and cardiovascular training	3. Trunk ROM within functional limits
			Gradually progress with exercise intensity and complexity to avoid sudden increases in stress on healing tissues	4. Functional capacity evaluation if appropriate	

Outcome measures and results

The patient's outcomes were assessed presurgery and at 14 weeks postsurgical rehabilitation using various measures, revealing notable improvements across all parameters. The VAS score for pain decreased from 7.5 to 2.5. RODI improved from 52% (severe disability, impacting travel, social life, and personal care) to 14% (minimal disability, allowing management of most daily activities with minimal treatment needed beyond advice on lifting, posture, and fitness). The sciatica bothersome index reduced from 16 to 5, while the MISI indicated significant improvement in urge urinary incontinence severity and quality of life, with scores dropping from 19 to five. Additionally RUIS score fell from 8 to 2, indicating a substantial reduction in symptoms. Table 8 gives the information about pre- and post-outcome measures.

**Table 8:** Outcome measure VAS: Visual analogue scale, lower scores indicate less pain discomfort; RODI: Revised Oswestry Disability Index (0%-20%: minimal disability; 20%-40%: moderate disability; 40%-60%: severe disability; 60%-80%: cripple; 80%-100%: bed-bound or symptom exaggerations); sciatica bothersome index: lower scores reflect better recovery from sciatica discomfort; MISI: Michigan incontinence symptom index, assesses type and severity of urinary incontinence and its impact on the quality of life (QOL); RUIS: revised urinary incontinence scale, this indicates severity of urinary incontinence

Outcome measure	Presurgery	Postsurgery/post rehabilitation (week 14)
VAS	7.5	2.5
RODI	52%	14%
Sciatica bothersome index	16	5
MISI	19	5
RUIS	8	2

## Discussion

This case report illustrates the successful management of McKenzie derangement VI with surgical intervention followed by a structured physiotherapy program. The patient’s significant postoperative improvements in pain reduction, functional capacity, and quality of life underscore the importance of a multidisciplinary approach to PIVD. The marked decrease in the VAS scores from 7.5 to 2.5, alongside the reduction in the RODI from 52% to 14% and the improvement in the sciatica bothersome index from 16 to 5, reflects the efficacy of both the surgical and rehabilitative interventions.

Surgical decompression and spinal fixation directly addressed the neural compression and instability caused by the herniated discs at the L4-L5 and L5-S1 levels. The surgical intervention successfully alleviated the mechanical pressure on the afflicted nerve roots, which is a critical factor in resolving the patient’s severe radicular pain and neurological deficits [14]. Postsurgery, the patient’s engagement in a tailored physiotherapy program was pivotal in her recovery. The program's phased approach facilitated the restoration of spinal mobility, strength, and functional independence, ultimately leading to a positive outcome. The rehabilitation protocol emphasized gradual progression through pain management, neuromuscular re-education, and functional restoration [15]. Early postoperative interventions focused on reducing pain and inflammation, while subsequent phases targeted the restoration of flexibility, stability, strength, and bladder control. The incorporation of cardiovascular training and activity-specific exercises further supported the patient’s functional recovery, allowing her to resume daily activities with minimal discomfort. Madera et al. emphasized the value of a structured surgical and phased rehabilitation plan, emphasizing gradual reactivation and strengthening, in achieving optimal outcomes for patients with PIVD [16]. Also Abu Raddaha et al. suggest the implementation of the Kegel exercise program led to significant improvements in both urinary incontinence and quality of life [17]. According to Alhakami et al., McKenzie and stabilization exercises proved more effective than conventional exercise programs in reducing functional disability in patients with chronic low back pain and listing correction [18].

The case also highlights the importance of preoperative assessments, including the modified Schober’s test, VAS, and RODI, in establishing baseline data for effective postoperative management. These tools were instrumental in measuring and monitoring the patient's development and making any adjustments to the rehabilitation plan [19].

Despite the positive outcome, the case underscores the potential complications of PIVD, such as chronic pain, neurological deficits, and the economic burden associated with disability and work loss [20]. Early intervention, accurate diagnosis, and a comprehensive treatment plan are essential in preventing such complications. Additionally, the success of the intervention relies heavily on patient adherence to the rehabilitation protocol, highlighting the importance of patient education and engagement.

Therefore, the combination of surgical decompression, spinal fixation, and a structured physiotherapy program effectively managed this case of PIVD [21]. The substantial improvements in pain, function, and overall quality of life emphasize the need for an integrated approach to treating herniated discs. Future studies should explore long-term outcomes and the potential benefits of early rehabilitation to optimize recovery in patients with cases of derangement.

## Conclusions

This case report demonstrates that a combined approach of surgical decompression, spinal fixation, and a structured physiotherapy program can effectively manage McKenzie derangement type VI with urinary incontinence. The patient experienced significant reductions in pain, functional disability, and urinary symptoms, underscoring the efficacy of a multidisciplinary treatment plan. The substantial improvements in the quality of life highlight the importance of integrated care for optimal outcomes in prolapsed intervertebral disc cases. This case reinforces the value of tailored rehabilitation and comprehensive management in achieving successful recovery and long-term functional improvement. Future research should focus on validating these findings and exploring early intervention benefits.
